# Hyperscanning EEG and Classification Based on Riemannian Geometry for Festive and Violent Mental State Discrimination

**DOI:** 10.3389/fnins.2020.588357

**Published:** 2020-12-16

**Authors:** Cédric Simar, Ana-Maria Cebolla, Gaëlle Chartier, Mathieu Petieau, Gianluca Bontempi, Alain Berthoz, Guy Cheron

**Affiliations:** ^1^Machine Learning Group (MLG), Computer Science Department, Université libre de Bruxelles (ULB), Brussels, Belgium; ^2^Laboratory of Neurophysiology and Movement Biomechanics, ULB Neuroscience Institute, Université libre de Bruxelles, Brussels, Belgium; ^3^Centre Interdisciplinaire de Biologie, Collège de France-CNRS, Paris, France; ^4^Department of Health, Medicine and Human Biology, Université Paris 13, Bobigny, France; ^5^Laboratory of Electrophysiology, Université de Mons-Hainaut, Mons, Belgium

**Keywords:** EEG, mental state, classification, machine learning, Riemannian geometry

## Abstract

Interactions between two brains constitute the essence of social communication. Daily movements are commonly executed during social interactions and are determined by different mental states that may express different positive or negative behavioral intent. In this context, the effective recognition of festive or violent intent before the action execution remains crucial for survival. Here, we hypothesize that the EEG signals contain the distinctive features characterizing movement intent already expressed before movement execution and that such distinctive information can be identified by state-of-the-art classification algorithms based on Riemannian geometry. We demonstrated for the first time that a classifier based on covariance matrices and Riemannian geometry can effectively discriminate between neutral, festive, and violent mental states only on the basis of non-invasive EEG signals in both the actor and observer participants. These results pave the way for new electrophysiological discrimination of mental states based on non-invasive EEG recordings and cutting-edge machine learning techniques.

## Introduction

Hyperscanning refers to the technique of simultaneous scanning, initially performed using fMRI, of participants’ brains who mutually interact in a controlled setting (Montague et al., 2002). The underlying neural basis is a consistent dynamical relationship between the interacting brains, which constitutes the essence of social interaction. Despite fMRI allowing high spatial resolution, this imaging technique cannot be employed during regular movements executed during social interaction in daily life. Hyperscanning EEG offers direct access to global electrical neural activity with an excellent temporal resolution that is necessary for assessing the brain dynamics of the interacting participants (Babiloni et al., 2006; Dumas et al., 2010; Barraza et al., 2019; Balconi et al., 2020). Moreover, EEG may characterize brain functions and states (Buzsáki and Draguhn, 2004). Hyperscanning EEG has been used in four specific domains of social interactions, joint action, shared attention, decision making, and affective communication (Liu et al., 2018). Although what is perceived as violent varies among societies and throughout human history (Elias, 1997), the effective recognition of festive or violent intent before the action execution remains advantageous for survival. When a violent intent emerges, individuals cognitively mobilize a particular mental state. In order to trace neural signals related to this mental state, we designed an experiment during which individuals perform the same kind of gesture—tossing a ball—but in a festive way and in a violent way.

Here, we present an experimental design in which two persons (one acting and the other observing) facing each other execute and observe, respectively, real movements with either festive, neutral, and violent intent. Both participants’ EEG, kinematics, and electromyographic signals were simultaneously recorded. Hyperscanning EEG during an actor-observer in a real face-to-face paradigm of social interaction coupled with kinematics has been previously introduced and investigated (Ménoret et al., 2014), showing that modulation of beta EEG oscillations in brain motor areas depended on the context (interaction vs. observation) and the role assignment (actor vs. observer). Also, EEG temporal dynamics have provided preliminary evidence of the ability to distinguish between the perception of kind, hostile, and non-interactive social intent inferred through visual scenarios on TV (Decety and Cacioppo, 2012; Wang et al., 2015) supporting that intentionality is the first input to moral computations (Decety and Cacioppo, 2012). In the last decade, new classification methods have been developed and applied mostly on brain-derived signals such as EEG (Wu et al., 2017; Lotte et al., 2018) and MEG (Fatima and Kamboh, 2017). The major area of interest was related to BCI application and less often to behavioral states identification, although previous work studied mental states directly linked to emotions (Kim et al., 2013; Schindler and Bublatzky, 2020) and social interactions (Kinreich et al., 2017; Liu et al., 2018; Czeszumski et al., 2020).

In this work, we used a classification algorithm on raw EEG trials of 10 couples of participants performing a repetition of festive, neutral, and violent throws. We hypothesized that EEG signals contain the distinctive information characterizing movement intent already before movement execution and that such distinctive information can be identified by state-of-the-art classification algorithms, among them one based on Riemannian geometry. Riemannian geometry classifiers have received growing attention in the last few years (Lotte et al., 2018), particularly due to their performance in international Brain–Computer Interface (BCI) competitions.

Here, we first illustrated the face-to-face hyperscanning condition before and during the execution of movement. Then, we justified the reason why the classification pipelines were applied on EEG periods occurring 1 s before the onset of movement. Then, we introduced the preprocessing algorithms and classification pipelines as well as the advantages of using Riemannian metrics when manipulating covariance matrices. The final classification results were then illustrated using a boxplot summarizing the performances of the classification pipelines applied on the EEG data from each of the 10 couples (actors and observers separately). We demonstrated that state-of-the-art classification pipelines can effectively discriminate between neutral, festive, and violent mental states using EEG signals from both the actors and observers. These results pave the way for new electrophysiological discrimination of mental states based on non-invasive EEG recordings.

## Materials and Methods

### Participants

The data were collected from 20 healthy right-handed [determined by the Handedness inventory (Oldfield, 1971)] male volunteers (24.5 ± 4.5 years old). Each participant gave informed consent to the experimental procedures, all of which were in accordance with the 1964 Helsinki declaration and its later amendments or comparable ethical standards.

### Experimental Design

This study has been inspired by the paradigm of Chartier et al. (2017) concerning the performance and perception of transitions from festive to violent gestures between two persons. The experiments were performed in the Jacques Lecoq theater school in Paris (Prof. Jos Huben). The gesture that was chosen was the simulation of the throwing of a ball to a partner. In this previous study, the actor’s gestures were analyzed when the throw was made in a neutral, festive, and progressively more and more violent mode. In these conditions, the main kinematic characteristics of the three categories of gestures were measured. In addition, from the recorded movie pictures of the throws, it was demonstrated that the intent behind actor’s gestures can be recognized by an observer even with morphing of the face taking away any possibility to recognize the emotional valence by the face expression. It has also been well demonstrated that bodily expression of emotions are well perceived without facial expression (de Gelder, 2016).

Here, we present a modification of this initial paradigm. In this new experimental protocol, two persons (one acting, the “*actor*,” and the other observing the “*observer*”) facing each other execute and observe, respectively, real movements with either festive, neutral, and violent intent.

Each couple of participants stood in an upright position facing each other and being separated by a distance of 4 m. The arms were at their sides. The actor held a foam ball (7 cm diameter) with the right hand. A LED light was fixed on the forehead of the observer. The verbal instructions to the actors were given in French to perform the following four tasks: resting, festive ball throw, violent ball throw, and neutral ball throw. The turn-on of the LED light placed on the observer’s forehead was the “go” signal administered in the four kinds of tasks:

• In the resting task, both participants remained standing at rest facing each other for 5 s during which the LED light on the observer’s forehead was turned on. This task was repeated 10 times.
• In the festive ball throw task, the actor was asked to perform 30 festive ball throws with the right upper limb aiming the LED light when it turned on and with increasing intensity of festivity following the indications of “execute a festive ball throw (10 times), a more festive ball throw (10 times) and an even more festive ball throw (10 times).”
• In the violent ball throw task, the actor was asked to perform 30 violent ball throws aiming the LED light when it turned on with increasing intensity of violence following the indications of “execute a violent ball throw (10 times), a more violent ball throw (10 times) and an even more violent ball throw (10 times).”
• In the neutral ball throw task, the actor was asked to perform 30 ball throws at slow (10 times), rapid (10 times), and as fast as possible (10 times) velocities and aiming the LED light when it turned on without any specific intent. This task was included in order to care about a possible velocity effect in the previous conditions.

**FIGURE 1:**
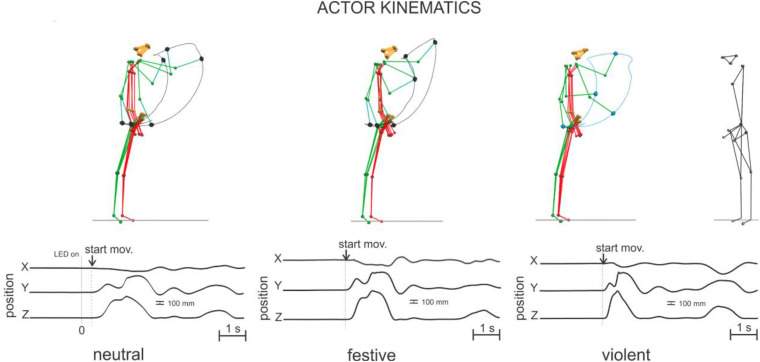
Actor’s stick diagram and 3D trajectory of the right head of ulna marker represented by the X (left and right), Y (forward and backward), and Z (up and down) coordinate components during the first ball throws of the neutral, festive, and violent tasks. All upper trace deviations correspond to the left, forward, and up directions, respectively. The observer stood facing the actor.

The festive and violent increasing gradation used was inspired by Gregory Bateson’s analyses (Bateson, 1955). The actor was asked to keep the same “type” of movement during the different trials and tasks (Figure 1). He tried all the tasks for familiarization before starting recordings. The observer was asked to not react to the foam ball throws in any condition. In order to facilitate the establishment of the different mental states, blocks of a same movement type were performed instead of intermixing trials of the different conditions.

During this procedure, the mental states of both participants were modified. For each of the three tasks, an instruction was given to the actor (neutral, festive, violent gesture) but the observer could hear the instruction and was, therefore aware of the mental state of the actor. Then, the successive repetitions of the same type of action reinforced, on one hand, the actor’s mental state related to his action by a modification of the motor networks from frontal and prefrontal cortex to supplementary motor (Scangos et al., 2013) and sensory-motor areas and more subcortical structures involvement, and, on the other hand, by the emergence of a “perceptual resonance” (Schütz-Bosbach and Prinz, 2007). For the observer, the repetition of the actor’s gesture induced, on one hand, an activation of the motor simulation (or “motor resonance”) networks involving the “mirror system” (Rizzolatti and Sinigaglia, 2007; Rizzolatti et al., 2014) and, on the other hand, an activation of the perceptual and motor imagery networks (Thirioux et al., 2010). Furthermore, a possible fatigue effect was avoided by the introduction of pauses between the different conditions.

### EEG Recordings

Both participants’ EEG, kinematics, and electromyographic signals were simultaneously recorded.

EEG data of the actors were recorded with 128 channels (ANT neuro system) at a sampling frequency of 2,048 Hz and with a resolution of 71.5 nV per bit. An active-shield cap using 128 Ag/AgCl sintered ring electrodes and shielded co-axial cables (5–10 electrode system placements) was comfortably adjusted to the participant’s head. In addition, electro-oculograms (EOG) (for horizontal and vertical eye movements) were recorded. EEG data of the observers were recorded with 32 channels (Brain Products Brainamp DC with actiCAP) with a resolution of 0.1 μV per bit at a sampling rate of 1,000 Hz. Common average reference was used for both recording systems.

**FIGURE 2:**
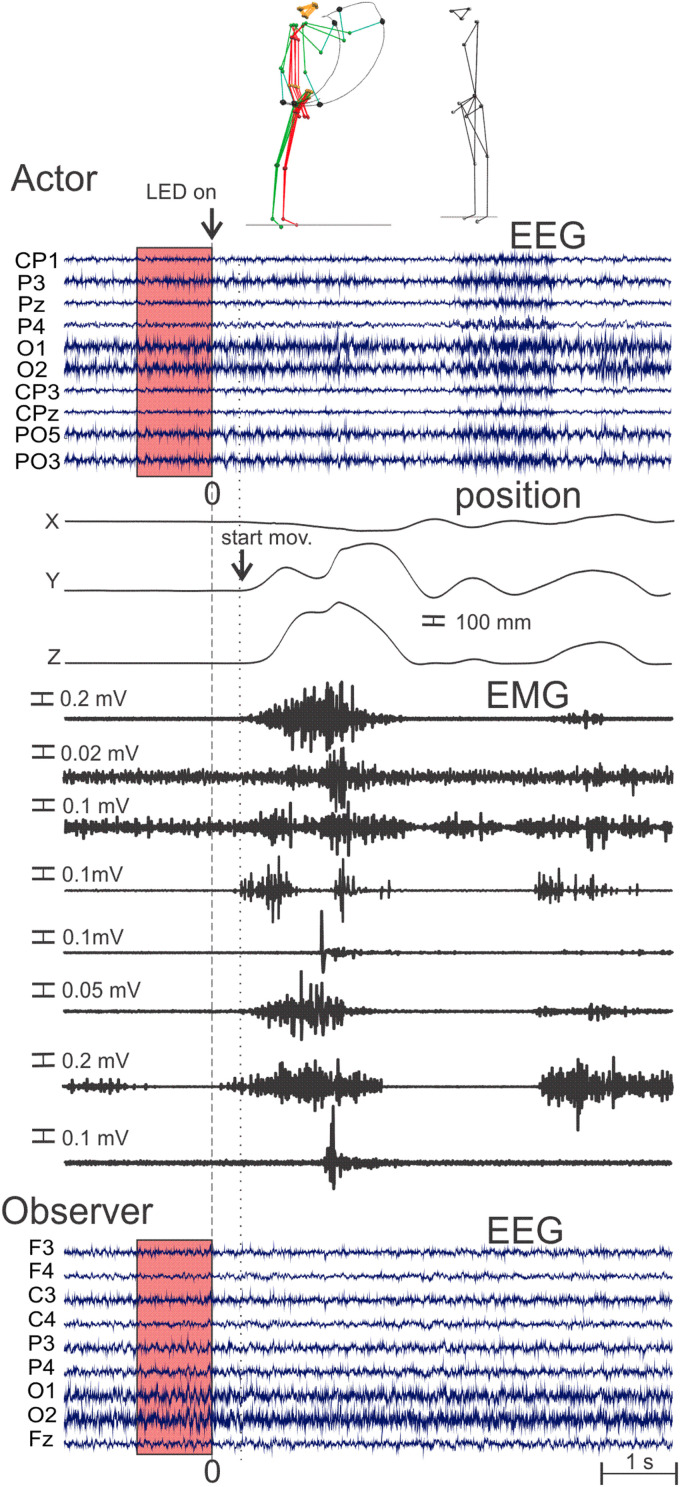
Simultaneously recorded biological signals for a representative couple during the first ball throw of the neutral task. The raw EEG signals of the electrodes selected for the classification are illustrated for both the actor (upper) and the observer (bottom). The position of the right head of ulna and the related raw EMG signals (from top to bottom: the anterior and posterior deltoids, posterior descending part of upper trapezius, short biceps, brachio-radial, pronator-teres, extensor carpi ulnaris, long head triceps brachii) are illustrated for the actor. Note that the rectangles colored in red contain the EEG signals’ epoch (of 1,000 ms preceding the LED light stimulus onset) provided to the classifier. The head of ulna 3D positions are represented by their X, Y, and Z coordinate components as in Figure 1.

Kinematics recordings were performed simultaneously (Figure 2) on both participants with VICON Motion Capture System with 10 cameras at 100 Hz sampling frequency. Passive infrared reflective markers were placed on the skin over nasion, tragus, acromion, lateral epicondyle of the right and left elbow, along an imaginary line between acromion and epicondyle on the right and left arms, over the head of right and left ulna, along an imaginary line between epicondyle and head of ulna on the right and left forearms, over the third metacarpal head on the right and left hands, right and left anterior superior iliac spines, right and left greater trochanter, right and left lateral epicondyle of the knees, along an imaginary line between greater trochanter and epicondyle of the knee on the right and left shanks, lateral malleolus, along an imaginary line between epicondyle of the knee and lateral malleolus over right and left legs, and over the second right and left metatarsal heads.

Electromyography (EMG) was also simultaneously recorded (Figure 2) with a sampling frequency rate of 1,000 Hz (Delsys Trigno Wireless System) on both participants with surface electrodes over the belly of the anterior and posterior deltoids, posterior descending part of upper trapezius, short biceps, brachio-radial, pronator-teres, extensor carpi ulnaris, and long head triceps brachii.

The four recording systems (ANT neuro systems, Brain Products, Vicon Motion Capture, and Delsys Trigno Wireless system) were synchronized with a common external TTL square signal delivered by an external signal generator to each recording system. This signal presented three rising and three falling edges at 1 Hz delivered at the beginning and the end of every task.

### EEG Data Treatment

The first offline data treatment consisted in eliminating the respective data portions recorded before the corresponding system received the first rectangular pulse of the synchronization signal.

Offline EEG data treatment was performed using the EEGLAB software (Delorme and Makeig, 2004). Initially, a 200 Hz low-pass filter, a 512 Hz resampling, and a 3 Hz high-pass filter were applied. Synchronous or partially synchronous artifactual activity (mostly blinks) was detected and rejected by independent component analysis (ICA). Baseline-corrected epochs were extracted from −1 to 3 s of the LED light turning on, i.e., the “go” signal. The signal-to-noise ratio (SNR) was computed on each electrode following Möcks et al. (1984), Turetsky et al. (1988), and Cheron et al. (2014). Formally, let *M* be the number of trials, *N* be the number of samples in a trial, and $\bar{X}$ denote the averaged signal over all trials; the total noise power $\sigma_{noise}^{2}$ and the total signal power $\sigma_{sig}^{2}$ can be defined as:

(1) $$\sigma_{\text{noise}}^{2}=\frac{1}{N\left(M-1\right)}\sum_{m=1}^{M}\left(\sum_{n=1}^{N}{\left(X_{m}\left(n\right)-\bar{X}\left(n\right)\right)}^{2}\right)$$

(2) $$\sigma_{sig}^{2}=\frac{1}{N}\sum_{n=1}^{N}{\bar{X}}^{2}\left(n\right)-\frac{1}{M}\sigma_{noise}^{2}$$

and the SNR can eventually be estimated by:

(3) $$SNR=\frac{\sigma_{sig}^{2}}{\sigma_{noise}^{2}}$$

For the following classification methodology, from all the biological signals of the actors and the observers, we selected epochs of EEG signals from −1 s to 0 s before the “go” indication and thus before movement preparation and execution (the selected epoch of a representative trial was represented by a red rectangle on the actor and observer’s EEG data in Figure 2).

### Classification Methodology

In order to verify that the classification was not based on the introduction of additional artifactual noise in one condition with respect to the others, we first built a naive classifier (Logistic Regression) solely based on SNR features.

Subsequently, in order to validate the possibility of effectively discriminating between different mental states, we applied two different state-of-the-art classification approaches. The first one used the Common Spatial Pattern algorithm (CSP) (Koles, 1991) improved with the robust estimation of covariance matrices manipulated with Riemannian geometry (Barachant et al., 2010a). For a binary classification task, the CSP algorithm optimizes spatial filters in a supervised way in order to maximize the variance of the filtered signals for one class and minimize their variance for the other class. Formally, let *E* ∈ *ℕ* be the number of electrodes, *N* ∈ *ℕ* be the number of time samples, *J* ∈ *ℕ* be the number of spatial filters, *W* ∈ R^E×J^ be the spatial filtering matrix used by the CSP algorithm, X_y_ ∈ R^E×N^ be the matrix representation of an epoch from class y, Σ^(*y*)^ ∈ *R*^E×E^ be the mean covariance matrix of class y, and *W*^*T*^*X*_*y*_ be the spatially filtered signal from class y. Given a binary classification task, the CSP algorithm first determines discriminative spatial filters *W* by the joint diagonalization of the two covariance matrices Σ^(1)^ and Σ^(2)^ as follows (Blankertz et al., 2008; Barachant et al., 2010a):

(4) $$\left\{ \begin{array}{c} W^{T}{\text{Σ}}^{\left(1\right)}W=D^{1}\\ W^{T}{\text{Σ}}^{\left(2\right)}W=D^{2}\\ D^{1}+D^{2}=I \end{array}\right.$$

Subsequently, the *n* most discriminative spatial filters (*n* being a parameter of the algorithm) determined on the training set are applied on EEG signals, and the variance of the resulting signal is then estimated. Since the variance of a signal band-pass filtered between the cutoff frequencies *f*_*L*_ and *f*_*H*_ is equal to the power of that signal in the [*f*_*L*_ − *f*_*H*_] frequency range, the CSP algorithm actually optimizes spatial filters in order to produce maximal power difference between the two classes. The output vector of the CSP algorithm is composed of the *n* variance estimations and will constitute the input of a classification algorithm such as a Logistic Regression. The multiclass generalization of the CSP algorithm is given by Grosse-Wentrup and Buss (2008).

The second approach using covariance matrices manipulated with Riemannian geometry have notably received growing attention in the last few years (Lotte et al., 2018), particularly due to their first-class performance in international BCI competitions (Congedo et al., 2017). In the present study, we used Riemannian geometry to manipulate covariance matrices of the filtered EEG signal, which is hypothesized to contain a simplified but discriminative representation of a mental state. Covariance matrices are symmetric positive definite (SPD) and do not lie in a vector space but in a convex cone (Moakher, 2005) called the Riemannian manifold. Thus, Riemannian metrics, i.e., distance and mean, should be preferred in order to manipulate these matrices accurately.

The Riemannian distance and mean are defined by Eqs (5) and (6), respectively:

(5) $$\delta_{\text{R}}\left({\text{Σ}}_{1},{\text{Σ}}_{2}\right)={∥\text{log}\left({\text{Σ}}_{1}^{-1/2}{\text{Σ}}_{2}{\text{Σ}}_{1}^{-1/2}\right)∥}_{F}$$

(6) $$\zeta \left(\Sigma_{1},\ldots ,\Sigma_{I}\right)=\underset{\Sigma \epsilon P\left(n\right)}{argmin}\sum_{i=1}^{I}\delta_{R}^{2}\left(\text{Σ},{\text{Σ}}_{i}\right)$$

where δ_R_, Σ ∈ *R*^*E*×*E*^, ∥.∥_*F*_, and *P*(*n*) denote the Riemannian distance, a covariance matrix estimated from *E* electrodes, the Frobenius norm, and the variety of symmetric positive definite matrices, respectively.

Additionally, for each point of the manifold, there is an associated tangent space where a scalar product is defined, and Barachant et al. (2013) showed that the Euclidean distance in the tangent space is a good approximation of the Riemannian distance on the manifold itself. This important finding means that tools and classification algorithms based on Euclidean geometry can be directly used in the tangent space without substantial loss in performance.

The projection operator from the Riemannian manifold to the tangent space at a reference point Σ_*ref*_ is defined by Eq. (7):

(7) $$\phi \left(\text{Σ}\right)={\text{Log}}_{{\text{Σ}}_{\text{ref}}}\left(\text{Σ}\right)={\text{Σ}}_{\text{ref}}^{1/2}\text{logm}\left({\text{Σ}}_{\text{ref}}^{-1/2}\Sigma \Sigma_{\text{ref}}^{-1/2}\right){\text{Σ}}_{\text{ref}}^{1/2}\;\left(7\right)$$

where Log_Σ_*ref*__ (Σ) denotes the logarithmic map (Barachant et al., 2013) of Σ with respect to Σ_*ref*_ and logm denotes the logarithm of a matrix. A good choice of Σ_*ref*_ is proposed by Barachant et al. (2013) to be the geometric mean of the whole set of covariance matrices and motivated by the observation from Tuzel et al. (2008) that the geometric mean is the point where the mapping on the tangent space leads to the best local approximation of the manifold.

### Classification Pipeline

High-density EEG typically records brain activity from at least 64 electrodes. However, from our own finding, manipulating covariance matrices estimated from a large number of electrodes might induce numerical errors that break their SPD property. Moreover, by using such large covariance matrices, the dimensionality of the feature space becomes significantly higher than the number of training data and thus increases overfitting and reduces the generalization accuracy of the classification algorithm (i.e., the curse of dimensionality). In such a situation, a common practice is a features selection procedure to reduce the number of features (in this case, the number of electrodes) in order to improve classification performances. In order to avoid biasing the classification results, the electrode selection procedure was applied using separate EEG recordings performed during the training period of the first couple preceding the effective hyperscanning performance during which the subjects executed a series of 30 throws in each condition. The same subsets of electrodes related to the actors and observers were subsequently used for the classification tasks of all 10 couples.

Firstly, we used separate data from the training period to empirically select electrodes that maximized the distance between class-conditional mean covariance matrices using a backward elimination method introduced in Barachant and Bonnet (2011). For this, we computed the average cross-validated binary classification accuracy with respect to the number of electrodes using the training data filtered with an IIR bandpass filter with cutoff frequencies at 1 and 20 Hz. This feature selection procedure resulted in a physiologically plausible choice of electrodes, namely, a common occipito-parietal subset (O1, O2, Pz, P3, and P4) for both the actors and observers and two different subsets, a centro-parietal subset (CP1, Cpz, CP3, PO3, and PO5) for the actors and a centro-frontal subset (C3, C4, F3, F4, and Fz), for the observers. Visual inspection of the selected electrode signals confirmed that no EMG contamination was present. We acknowledge that such feature selection procedure based on distances between class-conditional covariance matrices may slightly favor classification pipelines based on covariance matrices. Nevertheless, this potential bias is limited since the selected electrodes were well representative of the behavioral context linked to the task for both the actors and the observers. Moreover, such possible bias did not impact the main objective of this work, i.e., to demonstrate that state-of-the-art classification algorithm can effectively discriminate between neutral, festive, and violent mental states.

Subsequent to the electrodes selection procedure described here above, the EEG data files from the 10 couples were imported using the MNE 0.17 Python library (Gramfort et al., 2014). A zero-phase IIR bandpass filter with cutoff frequencies at 1 and 20 Hz was applied and epochs from −1,000 to 0 ms (0 ms being the “go” signal indicated by the LED lighting up) were extracted. In order to verify that the throw movement was not initiated before this “go” signal, the kinematic recording of the acting arm was visually inspected. At this stage, the dataset of each couple was composed of 180 matrices (30 for each class and for each role) of shape: 10 electrodes × 512 samples. The covariance matrix from each epoch is then estimated using the well-conditioned Ledoit-Wolf estimator (Ledoit and Wolf, 2004).

The following classification pipelines were then applied using the same subsets of electrodes:

• SNR with logistic regression: the SNR with logistic regression (SNR-LR) pipeline first estimates the SNR defined by Eq. (3) on each electrode. A logistic regression (LR) classifier is subsequently trained on the SNR values.
• Power spectrum density with logistic regression: the power spectrum density with logistic regression (PSD-LR) pipeline represents a simplistic approach that does just capture a part of the problem complexity and is not expected to yield state-of-the-art results. Nevertheless, this approach will serve as a robust baseline from which to evaluate more complex models. The power spectrum density (PSD) computes the log 10 of the average power in specific frequency bands (delta, theta, alpha, beta, and low-gamma) estimated using Welch’s method (Welch, 1967) on the epoch of EEG signal. The combined binned spectrograms from each electrode are flattened into a one-dimensional array of size 5 frequency bands × electrodes that represents an input to a LR classification algorithm.
• Common Spatial Pattern with logistic regression: the Common Spatial Pattern with logistic regression (CSP-LR) pipeline first applies the CSP algorithm on the raw EEG signals in order to optimize *n* spatial filters (in this work, we used *n=4*). The covariance matrices used internally by the CSP algorithm are estimated using the well-conditioned Ledoit-Wolf estimator (Ledoit and Wolf, 2004). A LR classifier is subsequently trained on the resulting features of the CSP algorithm.
• Covariance matrices with Minimum Distance to Mean: the covariance matrices with Minimum Distance to Mean (MDM) pipeline first estimates, for each epoch of EEG signal the corresponding covariance matrix using the well-conditioned Ledoit-Wolf estimator (Ledoit and Wolf, 2004). Subsequently, the Minimum Distance to Mean algorithm classifies covariance matrices directly on the Riemannian manifold.
• Geodesic filtering and covariance matrices with Minimum Distance to Mean: the geodesic filtering and covariance matrices with Minimum Distance to Mean (MDM-GF) pipeline first applies a geodesic filtering (Barachant et al., 2010b) in order to reduce the negative impact of noise on the distances between two covariance matrices. Subsequently, the MDM pipeline is applied on the output of the geodesic filtering.
• Projection on the tangent space and logistic regression: the projection on the tangent space and logistic regression (PTS-LR) pipeline first estimates, for each epoch of EEG signal, the corresponding covariance matrix using the well-conditioned Ledoit-Wolf estimator (Ledoit and Wolf, 2004). Then, each covariance matrix is projected on the tangent space of the Riemannian manifold using the projection operator defined by Eq. (7) and a LR classifier is subsequently trained on the projected covariance matrices.

The classification pipelines were implemented in the Python 3.6 programming language and use the NumPy (van der Walt et al., 2011), SciPy (Jones et al., 2001), scikit-learn (Pedregosa et al., 2012), and pyRiemann (Barachant and King, 2015) Python libraries.

## Results

First and foremost, it is important to highlight the fact that the classification pipelines were only applied on EEG signals occurring 1 s before the movement onset (see the red rectangle in Figure 2) in order to avoid the contamination of the EEG signals by muscular artifacts that would bias the classification performances.

### SNR Analysis

**FIGURE 3:**
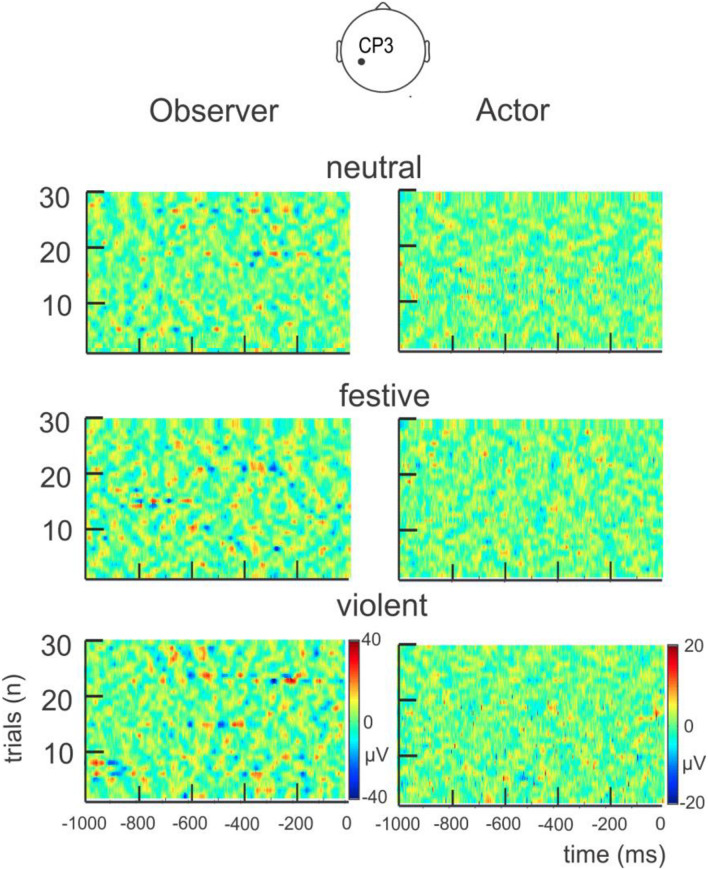
Variations of potential (μV) on CP3 channel during the selected epoch of 1,000 ms (preceding the LED light stimulus onset) provided to the classifier for the actor and the observer and for all the trials (30 throws per condition) of the neutral, festive, and violent conditions in one representative couple. Note that at first glance, there is not any consistent variation of potential for a condition.

**FIGURE 4:**
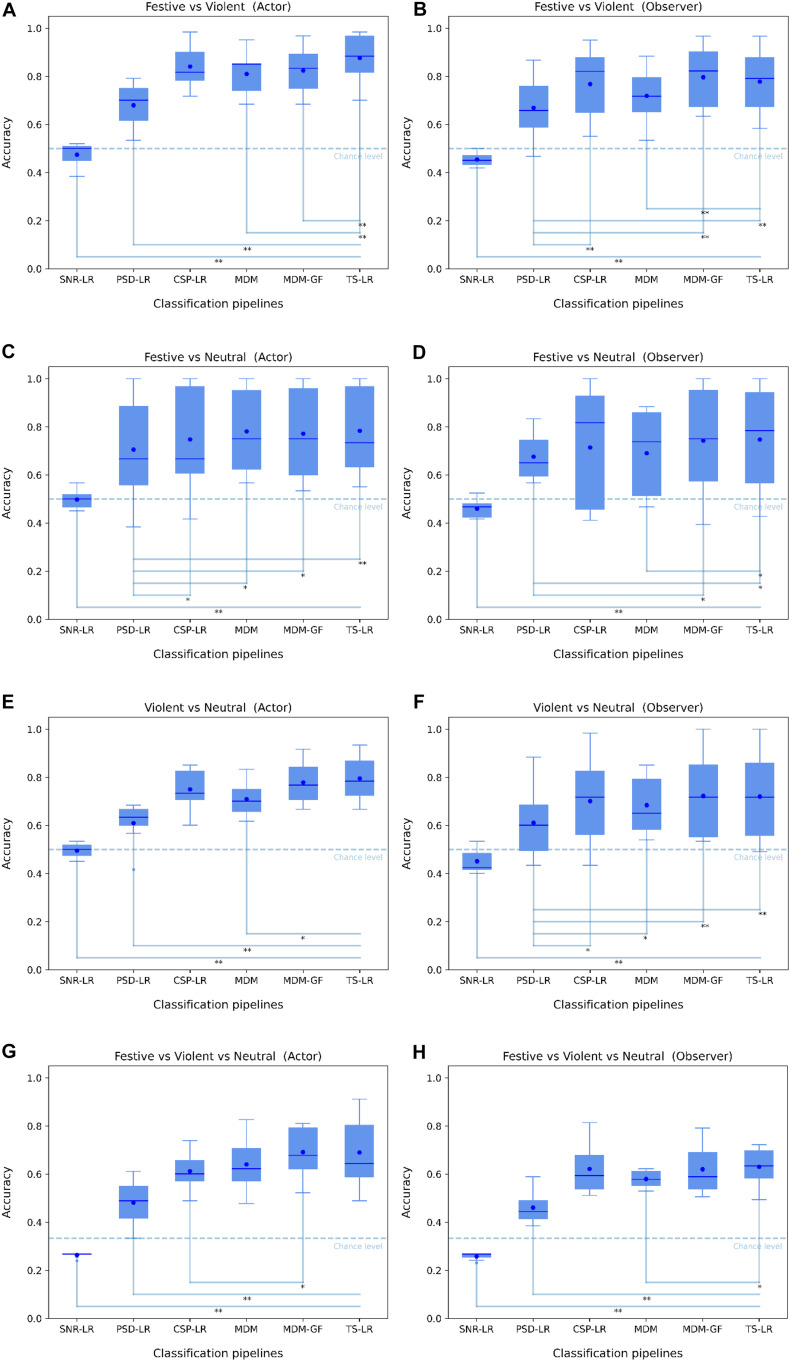
Boxplots illustrating the binary/multi-class classification accuracy (True Positives vs. All) of the SNR-LR, PSD-LR, CSP-LR, MDM, MDM-GF, and PTS-LR classification pipelines for the Festive vs. Violent, Festive vs. Neutral, Violent vs. Neutral, and Violent vs. Festive vs. Neutral mental states with regard to the participant conditions [Actor **(A,C,E,G)** and Observer **(B,D,F,H)**]. The horizontal dashed lines indicate the chance level. The blue hexagons indicate the mean classification accuracy values. Statistically significant differences between pairwise performances of two classification pipelines are represented using one or two asterisks when the *p*-value of the Wilcoxon signed-rank test is strictly below 0.05 or 0.01, respectively.

As a preliminary result, we verified that any specific changes in electrical potential (μV) was not obvious by simple visual inspection in any condition. For the 10 couples of subjects, the 30 trials of every condition (neutral, festive and violent) were plotted side by side separately for actor and observer. Figure 3 illustrates, for one representative couple, that the variation of electrical potential (μV) for every single EEG trial corresponding to every single epoch (1 s before the LED light turned on) before the throws for one representative electrode (CP3) cannot be visually discriminated. This first visual impression was then confirmed by a classifier based on SNR features that was not able to discriminate between the different mental states above chance level (see Figure 4).

**FIGURE 5:**
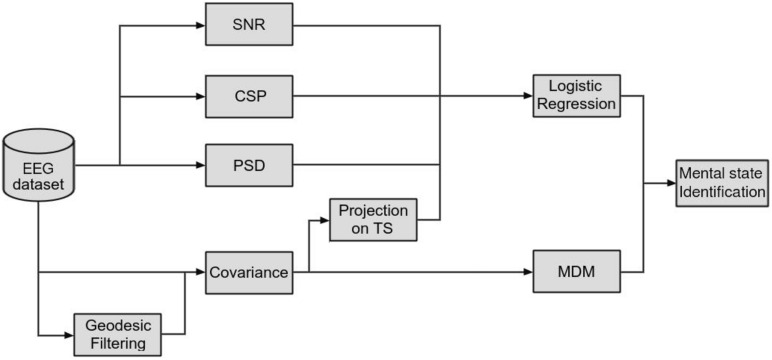
Illustrative summary of the classification pipelines. Each path from “EEG dataset” to “Mental state identification” represents a single and independent classification pipeline.

On the basis of the unsuccessful results of SNR classification, we turned to state-of-the-art classification pipelines (Figure 5) using CSP filtering and covariance matrices with Riemannian geometry.

### Benefits of Applying Riemannian Metrics

**FIGURE 6:**
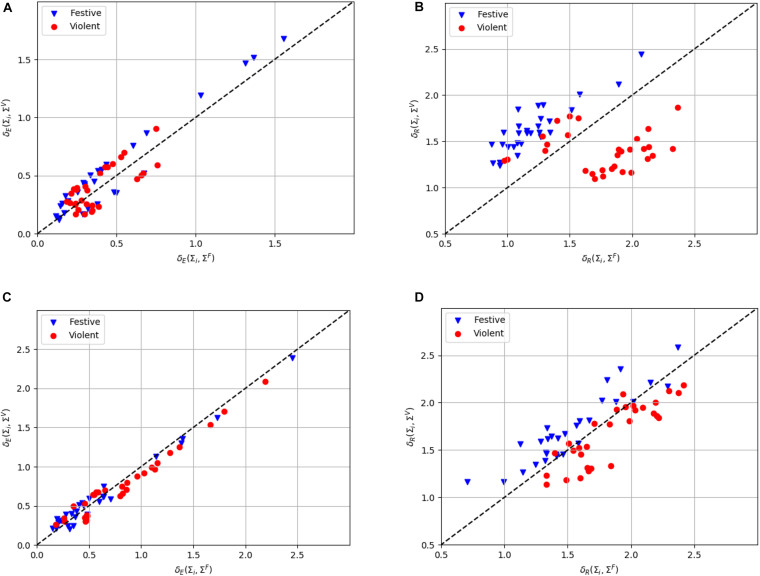
Distance to mean representation of the EEG data from the actor of the same representative couple as in Figure 3 using Euclidean **(A)** and Riemannian **(B)** distances and from the same representative observer using Euclidean **(C)** and Riemannian **(D)** distances. The Riemannian distance δ_R_ is defined in Eq. 5 and the Euclidean distance δ_E_ is defined as the Frobenius norm of the difference between the two covariance matrices. The horizontal (vertical) axis represents the distance δ(Σ_i_, Σ^F^) (δ(Σ_i_, Σ^V^)) between a covariance matrix Σ_i_ and the mean covariance matrix of the festive (violent) class Σ^F^(Σ^V^). The straight dashed line represents the function δ(Σ_i_, Σ^F^) = δ(Σ_i_, Σ^V^). Unlike Euclidean metrics, Riemannian metrics were able to convincingly separate the violent and festive classes.

In order to graphically illustrate the benefits of using Riemannian metrics and the projection on the tangent space when manipulating covariance matrices (estimated from the 10 selected EEG electrodes), we applied one procedure using the EEG data of one representative participant (the actor of the first couple). This procedure, called “distance to mean,” initially computed the mean covariance matrix of each class and subsequently represented the covariance matrix of an epoch on a graph by a single point whose coordinates corresponded to the distances between that covariance matrix and each mean covariance matrix. Figure 6 illustrates the benefits of using the Riemannian metrics to compute the distances between covariance matrices corresponding to the violent and festive EEG data set recorded from the actor of the first couple. In the Euclidean space, the experimental points associated with the two classes are overlapping (Figures 6A,C). In contrast, in the Riemannian space, the violent and festive data are better separable (Figures 6B,D).

### Classification Results

The boxplots of Figure 4 summarize the comparative analysis of all classification pipelines with respect to the mental state combinations. The datasets of all mental states and participants are perfectly balanced and the classification results for all pipelines were computed using a 10-fold cross-validation. We observed that statistically significant differences with a *p*-value < 0.05 computed using the Wilcoxon signed-rank test were found between the SNR-LR and all the other classification pipelines, regardless of the mental states or the conditions. This result combined with the SNR-LR classification accuracy under chance level confirmed our first impression that SNR features do not contain enough information to effectively discriminate between different mental states.

The PSD-LR results were also significantly lower than all the other classification pipelines in the Actor condition regardless of the mental states. In the Observer condition, the PSD-LR results were significantly lower than all the other pipelines except for the MDM in the Festive vs. Violent mental state (Figure 4B) and for the MDM and the CSP-LR pipelines in the Festive vs. Neutral mental state (Figure 4D). Even though there are few statistically significant differences between the CSP-LR and the Riemannian classification pipelines (MDM, MDM-GF, and TS-LR), the mean accuracies of the latter are slightly higher than the ones of the CSP-LR.

## Discussion

Understanding action intent of others involves matching the observed action to the internal representation of such an action built on one’s own multi-sensory-motor experience (Rizzolatti and Sinigaglia, 2007). Previously, hemodynamic-based neuroimaging brain studies have shown that the activated brain areas are different when understanding and judging a negative intent, which included the right amygdala, the temporoparietal junction, and hypothalamus (Sinke et al., 2010), compared to a positive intent, which included the right temporoparietal junction and the right dorsolateral prefrontal cortex (Yoder and Decety, 2014). In addition, fMRI (Vuilleumier and Pourtois, 2007; Bachmann et al., 2018; Engelen et al., 2018; Schönfeld and Wojtecki, 2019) and TMS (Borgomaneri et al., 2015; Engelen et al., 2015, 2018) studies demonstrated the existence of dynamical interactions between the amygdala, the inferior parietal lobule, and the ventral premotor cortex involved in the perception of emotions and the preparation of an action. The functional dynamical mechanisms underlying communication between brain areas have been approached with EEG, which, in this case, is particularly appropriate because it captures electrical brain oscillations that assess direct brain function with a millisecond precision. EEG recordings, while observing an intent inference task displayed on a screen, have shown that moral cognition processes occur as soon as at 200 ms where kind intent featured larger peak N2 amplitude component supported by left cingulate gyrus activity. Hostile intent featured later a larger peak P3 amplitude component supported by the left anterior cingulate cortex activity (Wang et al., 2015).

In this work, we propose a more realistic protocol where both the observer and the actor (of the violent, festive, and neutral ball throws) are real persons standing up and facing each other. This protocol allows access to the synchronized EEG dynamics, kinematics, and electromyographic activity of both participants. Although further investigation will be needed with a larger population to understand and characterize such EEG dynamics, kinematics, and muscular activity before, during, and after the movement, we demonstrated here that even without knowing the underlying dynamical mechanisms, classification algorithms can effectively discriminate between neutral, festive, and violent mental states. These successful classification results were obtained in both the actors’ and observers’ EEG signals of 10 couples during 1 s before the action, suggesting that festive and violent intent can be detected before the action. These results pave the way for new electrophysiological discrimination of mental states based on non-invasive EEG recordings.

### Design Considerations

The terms “festive” and “violent” can be understood differently depending on the context and interpretational meaning. The debate on whether and how linguistic data are a part of a complex interpretational structure and how they can be implemented in the mind of the participants remain largely unknown (Hagoort, 2020; Martin and Baggio, 2020). In this perspective, we propose a protocol inspired by Gregory Bateson’s analysis of double framing (Bateson, 1955). Bateson studied monkeys who were play-fighting and observed that this situation relied on two frames: the frame of the battle and the frame of the play. In the present design, the two participants are in the second frame of meta-communication since both the actor and the observer are aware of the general state imposed by the experimenter. This means that not only the posture and the gesture of the participant but also the imposed meta-cognitive communication may greatly contribute to the establishment of the required mental state. The fact that significant classification performances were obtained for the 10 couples of participants may indicate that the interpretational meaning of the “festive” vs. “violent” vs. “neutral” conditions were relatively well understood by all participants.

One of the major difficulties was to establish an experimental design able to provide a clear relationship between the intentional context, the mental states, and the behavioral output (Isoda, 2016). For this, we have approached the mental state by means of high-density EEG and the motor output by means of kinematics and EMG recordings. As it was reported that movement and EMG contamination of the EEG remain not satisfactorily solved (Castermans et al., 2014), we have focused the present mental state identification only during the epoch of 1 s before movement identification. During this preparatory period, the EMG artifact contaminations were not visible on the FFT spectrum and not identified with a classifier based on the SNR. In addition, no specific changes in electrical potential was detected by simple visual inspection. We may thus conclude that the present mental state classification was not based on EMG contamination.

### The Different Classification Methods and the Advantages of the Present One

The use of Riemannian geometry may at first be considered a mere mathematical sophistication. However, we here demonstrated that it has a profound impact on class separability and thus classification accuracy. This result strengthens evidence of the Riemannian geometry efficiency already reported in different scientific fields such as radar signal processing (Arnaudon et al., 2013), image classification (Tuzel et al., 2008), thermodynamics (Mausbach et al., 2018), morphogenesis (Hu et al., 2018), graph theory (Bakker et al., 2018) and BCI (Mayaud et al., 2016; Han et al., 2019; Rodrigues et al., 2019).

In order to verify our working hypothesis that the EEG signals characterized distinctively the festive, violent, and neutral mental states, we have systematically compared different classification methods currently used in the field and appropriate to the first explorative experiment carried out on a pair of synchronized EEG recordings of 10 observers and 10 actors. Our results demonstrate that state-of-the-art classification algorithms based on Riemannian geometry (MDM, MDM-GF, and PTS-LR) or Common Spatial Pattern (CSP-LR) are able to effectively discriminate between mental states (reaching a cross-validated classification accuracy of 0.88 for the Festive vs. Violent states) and provide significantly better performance with respect to classifiers based only on SNR or PSD. We showed that the use of the variance–covariance alone was unable to effectively discriminate between the three mental states, which indicates that the Riemannian geometry is crucial for neural signal discrimination based on covariance matrices. Although classifiers based on the CSP algorithm perform slightly worse than the Riemannian methods in international BCI competitions (Lotte et al., 2018), the CSP-based classifier has here produced comparable performances. It is also interesting to mention that, in spite of the fact that both participants are aware of the Festive or Violent required condition, EEG classification performances were better for the Actor, which may indicate that the motor-action preparatory processes propagate to the EEG signals and thus play a role in the identification of the mental state.

The assumption that the EEG signal observed prior to the action is necessarily a sign of intent must be met with some caution, as there could be other equally plausible explanations, such as the prediction of an incoming “known” stimulus, which cannot be excluded in the absence of “blinding.” Further studies could potentially provide further evidence for or against the specific mechanism proposed in this work.

Interestingly, state-of-the-art classifiers used in this study were able to achieve significant discriminability using a limited number of trials. Such desirable characteristics is paramount to avoiding side effects such as fatigue, habituation, or loss of awareness induced by too much repetition of the same behavior and related mental state induction. Nevertheless, these results are also relevant for future BCI applications where limited signal acquisition is a major constraint to train a functional classification algorithm.

## Data Availability Statement

Requests to access the datasets should be directed to GCe, gcheron@ulb.ac.be.

## Ethics Statement

All experimental protocols were approved by the Ethic comity of Université Libre de Bruxelles, CHU Brugmann and conducted in conformity with the European Union directive 2001/20/EC of the European Parliament. The patients/participants provided their written informed consent to participate in this study.

## Author Contributions

AB and GCa conceived the original idea. CS, A-MC, and GCe designed the experiment. AB, CS, A-MC, and GCe performed the experiment. CS and GB performed the data analysis, GCe and CS wrote the manuscript. AB, GCa, A-MC, and GB contributed to the writing. All authors contributed to the article and approved the submitted version.

## Conflict of Interest

The authors declare that the research was conducted in the absence of any commercial or financial relationships that could be construed as a potential conflict of interest.
